# Multidisciplinary, multicomponent interventions to reduce frailty among older persons in residents of residential care facilities: a scoping review

**DOI:** 10.1186/s13643-024-02576-3

**Published:** 2024-06-10

**Authors:** R. C. Ambagtsheer, M. J. Leach, L. M. O’Brien, J. Tyndall, J. Wardle, J. Beilby

**Affiliations:** 1 Torrens University Australia, Adelaide, SA 5000 Australia; 2 Faculty of Health, National Centre for Naturopathic Medicine, Southern Cross University, Lismore, NSW Australia

**Keywords:** Frailty, Residential care facilities, Model of care, Multidisciplinary, Multicomponent

## Abstract

**Background:**

Frailty reduction and reversal have been addressed successfully among older populations within community settings. However, these findings may not be applicable to residential care settings, largely due to the complex and multidimensional nature of the condition. Relatively, few attempts at frailty prevention exist in residential settings. This review aims to identify and describe best practice models of care for addressing frailty among older populations in residential care settings. This research also sets out to explore the impact of multidisciplinary health service delivery models on health outcomes such as mortality, hospitalisations, quality of life, falls and frailty.

**Methods:**

A scoping review of the literature was conducted to address the project objectives. Reference lists of included studies, bibliographic databases and the grey literature were systematically searched for literature reporting multidisciplinary, multidimensional models of care for frailty.

**Results:**

The scoping review found no interventions that met the inclusion criteria. Of the 704 articles screened, 664 were excluded as not relevant. Forty articles were fully assessed, and while no eligible studies were found, relevant data were extracted from 10 near-eligible studies that reported single disciplines or single dimensions rather than a model of care. The physical, nutritional, medicinal, social and cognitive aspects of the near eligible studies have been discussed as playing a key role in frailty reduction or prevention care models.

**Conclusion:**

This review has identified a paucity of interventions for addressing and reducing frailty in residential care settings. High-quality studies investigating novel models of care for addressing frailty in residential care facilities are required to address this knowledge gap. Similarly, there is a need to develop and validate appropriate screening and assessment tools for frailty in residential care populations. Health service providers and policy-makers should also increase their awareness of frailty as a dynamic and reversible condition. While age is a non-modifiable predictor of frailty, addressing modifiable factors through comprehensive care models may help manage and prevent the physical, social and financial impacts of frailty in the ageing population.

**Supplementary Information:**

The online version contains supplementary material available at 10.1186/s13643-024-02576-3.

## Background

Population ageing has considerable implications for global health, contributing to an increased prevalence of chronic disease, falls, dependency and frailty [1, 2]. Frailty is a clinically recognised syndrome reflecting a state of vulnerability to stressors. While age is a recognised non-modifiable predictor of frailty, it represents only one of a multitude of contributing factors [3–5]. Many of these determinants are modifiable (e.g. physical inactivity, malnutrition, depression, social isolation and polypharmacy), suggesting that frailty is a manageable, and possibly preventable condition [3–5]. Frailty is widely recognised as being a multidimensional concept, involving both physical and psychosocial factors; however, there is no single accepted standard measure, with different instruments (e.g. the frailty phenotype, frailty index) considered suited to specific applications [6].

Numerous studies conducted within community settings have shown promising results with respect to frailty reduction and reversal [3, 4, 7, 8]. However, these findings may not necessarily translate to noncommunity settings, such as residential aged care [9]. Unfortunately, attempts to address frailty within residential care settings have been comparatively few, resulting in an evidence gap in frailty prevention [10].

Many authorities have advocated for a shift away from uni-disciplinary, singular interventions for frailty prevention and management, to innovative models of care [11–13]. A model of care defines the principles and components of how health services are arranged and delivered for best practice and positions these within an implementation and evaluation framework [14, 15]. Models of care also can help broach evidence gaps and facilitate the provision of resource efficient, individualised care, particularly when the condition is complex and multidimensional [16, 17]. Accordingly, a multidisciplinary model of care would be apt for addressing frailty in residential care settings [5, 7, 18].

The aim of this review is to identify and describe best practice models of care for addressing frailty in residential care settings among residents aged 65 + years. A secondary objective of the review is to explore the impact of these models of care on pertinent patient outcomes (i.e. mortality, hospitalisations, quality of life, falls, frailty) [19]. It is envisaged that the findings of this review will help guide health service delivery in residential care settings to improve the health outcomes of those living with frailty.

## Methods

### Study design

Frailty is a broad topic with multiple domains to which differing study designs might be applicable; consequently, a scoping literature review was conducted using the framework of Arksey and O’Malley [20]. The review was reported in accordance with the Preferred Reporting Items for Systematic Reviews and Meta-Analyses (PRISMA) extension for reporting Scoping Reviews (PRISMA-ScR) [21].

### Data sources and search strategy

The search strategy was designed in consultation with an academic librarian and the research team. A preliminary search was conducted by the librarian within PubMed to refine the search terms: *frail* or *model of care* or *nursing homes* in the title/abstract or *frailty* or *nursing home* or *aged* or *aged 65 and over* in the Medical Subject Headings (MeSH) or *elder* or *older people* or *older adult* or *senior* or *retire* or *geriatric* as a TIAB (free-text term) search in the title/abstract. Varying the search terms allowed for variations in international usage of key words [20–22].

The same combination of MeSH terms and keywords was used to perform systematic searches for eligible articles in PubMed, CINAHL, Scopus and the Cochrane Library for recency from January 01, 2010, to August 24, 2022 (i.e. date of search). Search syntax for PubMed is provided in Appendix A, and search syntax for other data bases is comparable. We also conducted modified versions of the same search strategy for unpublished literature in Social Care Online, TRIP database, Health Evidence Canada, Internet Scholar Archive, PROSPERO, AHRQ, OpenGrey and the Grey Literature Report and for trials in WHO International Clinical Trials Registry Platform (ICTRP) and ClinicalTrials.gov. EThOS, DART Europe and Trove were searched for dissertations. An iterative approach was adopted to refine, adjust and refocus the search strategy in the light of sets of preceding results which optimised the identification of eligible studies. Professional organisations and associations (e.g. Association of Gerontology & Geriatrics, Frailty Forum [NSW] and Canadian Frailty Network) were consulted, and the *Journal of Nursing Home Research* and the *Journal of Frailty and Aging* were hand searched.

The reference lists of included literature were manually scanned to identify additional references. The search was limited to articles published in English.

### Study selection

#### Selection criteria

Delphi studies, observational studies, clinical trials, systematic reviews and meta-analyses describing models of care for addressing frailty in residential care settings were eligible for inclusion in this review. Clinical guidelines and position papers/statements from peak bodies were also considered. In terms of measuring the impact of these models of care, studies using qualitative, quantitative and/or mixed methods designs were considered.

Participants aged 65 + years living in residential care settings, including nursing home settings and/or long-term care facilities, in any country were chosen as the target population as this age group has been found to have considerable levels of frailty along the frailty continuum [7].

Studies reporting a multidisciplinary, multidimensional model of care that sets out to reverse or treat frailty were included. Within the context of this review, we defined a model of care as envisioning and defining the way integrated health services are delivered, facilitating the provision of timely, equitable and individualised care, particularly when the condition is complex and multidimensional along a continuum [14, 17]. To meet the multidisciplinary criterion, professionals from more than one discipline needed to have been specified to have administered the intervention. The multidimensional criterion required that the model of care address more than a single dimension of health and well-being (for example interventions addressing both physical and social dimensions were eligible for inclusion).

The primary outcome of this review was frailty, as identified by the authors of the individual studies. Secondary outcomes were changes in pertinent patient outcomes such as mortality, hospitalisations, quality of life and falls.

### Data extraction and quality assessment

Results of the search were imported into Covidence software [22]. Title and abstract screening was completed using the accelerated approach, with LO screening all citations and RA screening 10% of randomly selected citations. Two reviewers (R. A., M. L.) independently screened all full-text articles, with a third reviewer (L. O.) allocated to resolve disagreement.

Data from included studies were expected to be charted by one reviewer (L. O.) and verified by two reviewers (R. A., M. L.) using a customised charting form. This form aimed to capture the following data: author/date, research gap, aims/objectives, country, population, intervention, comparator, outcome measure, results, limitations and enablers/barriers to the research.

In the event the screening process resulted in an empty review, the reviewers agreed a priori to extract data on near-eligible studies (i.e. studies meeting all selection criteria, except that they reported a single dimension/single discipline rather than a model of care). The purpose of this step was to identify potential components of a model of care for frailty management and/or prevention that may help inform future research, policy and/or practice.

### Data synthesis

Given the descriptive nature of the review question, charted data were synthesised in narrative form.

## Results

The search identified 752 articles. Following the removal of 48 duplicates, 704 articles underwent title and abstract screening. Of these, 664 articles were excluded as not relevant.

The objective of this review was to identify best practice models of care for addressing frailty among older residents (aged 65 + years) of residential care facilities. While no eligible studies were found, a number of near eligible (i.e. studies reporting single disciplines/single dimensions rather than a model of care) were identified [23, 24]. These data are presented in Table 1 as recommended by Lang et al.’s (2007) [25] seminal paper on empty reviews. The data in Table 1 represents populations from five countries within Asia, Europe and the United States, with study sample sizes ranging from 18 to 248. The duration of the near-eligible studies in Table 1 varies from 2 and 3 months, to half a year and a year. One non-randomised controlled trial is listed in Table 1, resulting in no significant improvement for gait speed outcome.

**Table 1:** Summary data from excluded studies (full-text round)

**Study ID**	**Population**	**Intervention**	**Frailty outcome**	**Results**	**Exclusion reasons**^**a**^
Courel-Ibáñez (2022) (#457)	Spain *N* = 24 87.1 ± 7.1 years (58.3% female)	24-week randomised trial. Test group completed 24 weeks supervised Vivifrail multicomponent exercise training followed by 6 weeks of detraining	Vivifrail classification	Frailty reversed in 36% of participants	XML
Feng (2021) (#191)	China *N* = 18 65 + years, mean age 85.94 (*SD* 3.17) years (94.4% female)	3-month single group, pre-/post-test (two-phase delivery) study. Phase 1: participants received movement instruction and practice for 4 weeks. Phase 2: participants undertook the Otago exercise programme three times/week for 36 sessions	Fried frailty phenotype	The frailty score decreased significantly (*p* < 0.05) in the intervention group	XML
González-Bernal (2021) (#174)	Spain *N* = 80 75 + (84.2 ± 8.7) years (*n* = 45 females)	8-week longitudinal, pre-/post-test study. The test group attended 20 sessions of 40-min Wii virtual reality video games	Gait speed test Fried frailty phenotype	Walking speed results test group compared to control group (− 6.42 ± 8.83% vs. 0.12 ± 4.51%) Frailty levels positively influenced in the test group	XML XMD
Grubbs (2020) (#551)	USA *N* = 25 65 + years	12-week non-randomised controlled trial. Participants performed whole body vibration training sessions twice/week	Gait speed test	No significant improvement	XML
Liu (2022) (#491)	China *N* = 146 75 + years, mean age 80.74 ± 2.89 years (70.37% female)	12-month single-blinded, parallel group randomised controlled trial. Intervention group performed individually tailored multicomponent aerobic exercises for 40-min five times/week	Fried frailty phenotype	Phenotypic frailty score (β3 = − 1.40, *P* < 0.001) decreased significantly in the intervention group	XML
Meng (2020) (#117)	China *N* = 66 ≥ 60 years, mean age 81.8 years (71% female)	12-week quasi-experimental trial. The intervention group participated in 40-min sessions of group dancing at mild to moderate intensity three times/week	Fried frailty phenotype	Frailty decreased over time in the dance group (*P* = 0.002)	XML XMD
Rezola-Pardo (2019) (#263)	Spain *N* = 85 ≥ 70 years	3-month single-blinded, randomised controlled trial. Test group performed 1-h individualised and progressive strength and balance moderate intensity exercise training sessions twice/week	Fried frailty phenotype Tilburg frailty indicator	Only the multicomponent group significantly reduced Fried frailty score (*P* < 0.05)	XML
Roughead (2022) (#461)	Australia *N* = 248 Median age 87 years	12-month open-label, parallel randomised controlled trial. Intervention group received a pharmacist review of their resident care record and medicine chart, including pharmacist meeting, every 8 weeks	Frailty index	No statistically significant difference for change in frailty between groups (mean difference: 0.009, 95% *CI*: − 0.028, 0.009, *P* = 0.320)	XML XMD
Sahin (2022) (#545)	Turkey *N* = 72 ≥ 65 years	12-week randomised controlled trial. Intervention group performed 45 min of individualised Otago strength and balance exercises 3 days/week, plus a walking programme in the other 3 days of the week	Edmonton Frail Scale (EFS)	Significant differences between the intervention and control group mean EFS (*P* = 0.018)	XML
Theou (2019) (#118)	Spain *N* = 50 65 + (75.3 ± 7.3) years (70% female)	13-week secondary analysis of a placebo-controlled double-blind randomised study. Intervention group received daily inulin and fructooligosaccharide formulation	Frailty index (FI)	Among the 28 participants in the intervention group, FI levels were reduced for 25 people (0.20 ± 0.08, *P* < 0.001); 5 of them had an FI reduction greater than 0.03. The moderately/severely frail participants (*FI* > 0.3, *N* = 5) had the greatest reduction in their FI (0.04 ± 0.01)	XML

^a^ *XML*, not multidimensional; *XMD*, not multidisciplinary

**Fig. 1:**
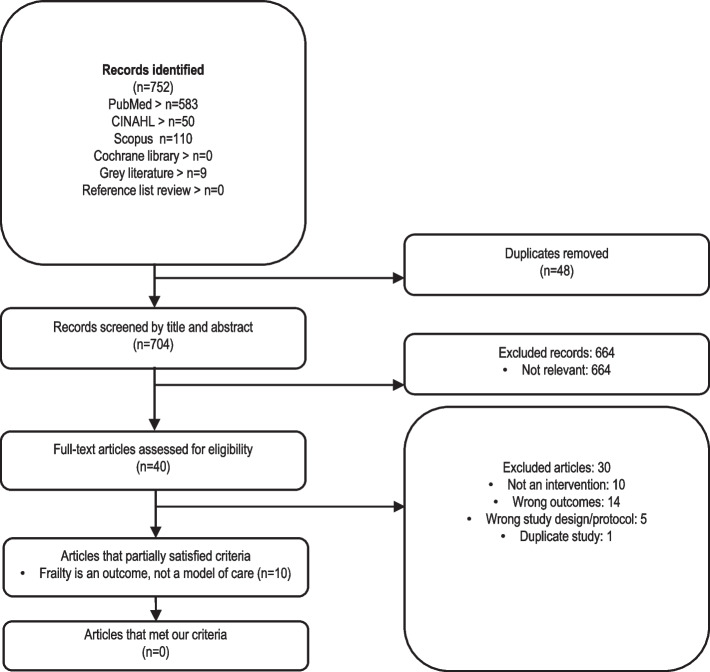
PRISMA diagram of the scoping review literature search and selection process

Although many of the excluded studies did involve multidisciplinary teams, most were excluded due to a single intervention focus — be that physical [26–33], pharmaceutical [34] or nutritional [35]. Study designs varied, inclusive of randomised and non-randomised controlled trials and quasi-experimental and single group pre-post studies. While two studies did report interventions that comprised an additional dimension beyond a physical component (e.g. cognitive or psychological elements), these studies were ineligible for inclusion within our review as they were conducted by a professional from a single discipline only (e.g. physiotherapy) or did not provide sufficient information on this aspect of the study [32, 33] (Fig. 1).

## Discussion

Despite more than a decade of discourse advocating multidisciplinary models of care for frailty [5, 7, 17], our review found no studies describing such models for persons aged 65 + years living in residential care settings. There are several potential explanations for our empty review, including the widely recognised difficulties associated with implementing multidisciplinary care [36], the relatively recent emergence of the research topic and the defined scope of the review [24, 37]. Further, the residential aged care sector on a global basis continues to face significant workforce and fiscal challenges [38, 39] that may make the goal of multi-disciplinary, team-based care aspirational rather than achievable in many respects. A more patient-centric agenda that incorporates multidisciplinary interventions is needed [5, 7, 18].

Frailty remains a nascent topic of enquiry within the broader discipline of geriatrics and gerontology [4, 7], with the first clinical conceptualisation of frailty dating back to 2001 [8]. Intervention studies on the whole have been few and tend to be overwhelmingly concentrated within community settings rather than residential care facilities. Residential care populations have likely been deprioritised as subjects of frailty research for several reasons. One is a consequence of pervasive ageism within society that continually de-emphasises older people in general, and older people living within residential care settings specifically, as a worthy focus for interventional research [4, 7]. Emergent research also suggests that clinicians typically recognise frailty only in its advanced stages, at which point the intention of care provision has largely been crisis oriented and geared towards stabilisation rather than towards preventative or restorative care [1, 5]. Consequently, residential care populations may be perceived as being past a viable point of intervention. The validity of existing frailty outcome measures is an additional consideration. Despite the availability of a multitude of frailty screening and assessment instruments, most have been developed and validated among community-dwelling rather than residential care populations [3, 40].

With respect to the scope and inclusion criteria for the review, given the global emphasis on holistic models of care as being most appropriate to the care of older people [41, 42], we did not anticipate that our requirement for included interventions to be both multi-disciplinary and multi-dimensional would pose a significant problem. However, the studies we did identify for potential inclusion were, without exception, excluded at the full-text screening stage because they were uni-disciplinary and/or uni-dimensional, despite the syndrome of frailty being widely acknowledged as a complex and multidimensional phenomenon [3–5]. This is in stark contrast to other complex, multidimensional conditions such as diabetes and chronic non-specific low back pain, where multidisciplinary, biopsychosocial models of care are both advocated and clearly defined [43, 44].

The profound focus on addressing physical predictors of frailty (e.g. strength, balance), with very little attention afforded to psychological, social or environmental predictors, is in large part attributable to the frailty instrument selected as the study outcome measure. Of the 10 near eligible studies, the majority (70%) applied a purely physical construct to measure the frailty outcome, generally the Fried frailty phenotype [45], which does not readily lend itself to a multicomponent intervention. Of the three remaining studies, all adopted a unidimensional focus (i.e. medication management, exercise or nutrition) largely determined by the uni-disciplinary background of the study administrators. To some extent, these studies propagate a misguided notion that frailty is largely a physical condition responsive primarily to physical interventions; preserving such a notion may only serve to perpetuate unmet health care needs in people living with frailty.

Within the context of the current study, a frailty model of care could be conceptualised as an evidence-based, best practice approach to the provision of services and care for older people living with frailty in residential care settings. Drawing upon the evidence base presented in Table 1, along with recent comprehensive summaries of the current state of knowledge within the field, it is possible to outline what such a model might look like. In essence, a frailty model of care should be able to map the individual needs of a resident (i.e. modifiable biological [malnutrition, poor strength], social [social isolation] and psychological [anxiety, depression] determinants of frailty) against an appropriate multidisciplinary team (e.g. dietician, physiotherapist, psychologist) that have the competency to administer suitable evidence-based interventions in a coordinated, efficient and safe manner.

The absence of a body of interventions directed towards addressing frailty avoidance or reduction in residential care settings points to some important recommendations for future research, policy, practice and education. Clearly, there is a need for more large-scale, high-quality, multidimensional frailty intervention studies to be conducted within residential care settings, along with appropriate investment in research and innovation funds. Additionally, more research needs to be devoted towards developing and validating multidimensional frailty screening and assessment instruments that are appropriate for residential care populations. To be able to effectively intervene in frailty, we need to be able to measure it first. It is also clear that more education and training need to be directed towards raising awareness of frailty as a dynamic and potentially reversible condition, among both health and aged care service providers and policy-makers [46]. A continuing perception that frailty can only be managed, rather than targeted for intervention, will deny older residents of residential care facilities significant potential improvements in quality of life. Residential care residents (and older people in general) also need to be acknowledged as a population worthy of clinical intervention, rather than viewed as being on an inevitable trajectory of worsening health status culminating in end-of-life care. The World Health Organization has in recent times spearheaded this effort through its extensive global ageism campaigns, but there is much more to be done [47].

Our finding of an empty review might suggest the presence of a considerable research gap in the literature; however, this should be considered in light of the study limitations. Even though the review was undertaken using a comprehensive and sensitive search strategy, developed in consultation with a senior academic librarian specialising in the health field, we were unable to identify best practice models of care for frailty within residential care settings. This finding was due in large part to our determination that included interventions should be both multidimensional and multidisciplinary, which could be viewed as a limitation due to the relatively narrow inclusion criteria for the model of care. However, as we have previously discussed, such considerations are considered critical inclusions in the best practice models of care advocated for older people in the future. Consequently, while recognising the concomitant risks, a decision was made to preference specificity over sensitivity in searching the literature. A further limitation of this review was that the search was limited to articles published in English, meaning that potentially relevant studies may have been overlooked. As well as English-language issues, the terms “multi-disciplinary”, “multi-dimensional” and “model of care” may have different terminologies even within English due to national or regional preferences. While all efforts were made to be inclusive (e.g. through full-text screening and in-depth review of articles), a lack of standardised terminology and keywords may have resulted in some missed results.

## Conclusion

Despite extensive calls for frailty among older people to be treated using a person-centred, holistic care planning approach that incorporates multiple d of health and providers from multiple disciplines, our systematic review did not identify any interventions within residential care settings that met this description. Excluded interventions had either a single component that was primarily physical or, where multi-dimensional, was administered by only a single discipline. No interventions met the ideal of a multidimensional, multidisciplinary model of care. Although the utility of empty reviews is sometimes questioned, our review points to both (1) an overarching need for more comprehensive interventions to be developed and (2) an extensive gap in the literature with regard to this topic. There is a clear need for person-centred, multidimensional, multidisciplinary models of care suited to addressing frailty within residential care facilities to be tested in future studies.

### Supplementary Information

Additional file 1: Appendix A: Search syntax for PubMed

## Data Availability

Not applicable.
