# Supplementary material for: Multidisciplinary, multicomponent interventions to reduce frailty among older persons in residents of residential care facilities: a scoping review
Source: Syst Rev. 2024 Jun 10;13:154. doi: 10.1186/s13643-024-02576-3 (PMC11163739; doi:10.1186/s13643-024-02576-3)
Supplement: Supplementary file 1 — Additional file 1: Appendix A: Search syntax for PubMed [file 13643_2024_2576_MOESM1_ESM.docx]

**Appendix A Search syntax for PubMed**

("Aged"[MeSH Terms] OR "aged, 80 and over"[MeSH Terms] OR "elder*"[Title/Abstract] OR "older people"[Title/Abstract] OR "older adult*"[Title/Abstract] OR "senior*"[Title/Abstract] OR "retire*"[Title/Abstract] OR "geriatric*"[Title/Abstract]) AND ("Nursing Home"[Title/Abstract] OR "Nursing Homes"[MeSH Terms]) AND ("Frailty"[Title/Abstract] OR "Frailty"[MeSH Terms])

English language 2010-2022.

576 results.

The following resulted in 893 articles

("Aged"[MeSH Terms] OR "aged, 80 and over"[MeSH Terms] OR "elder*"[Title/Abstract] OR "older people"[Title/Abstract] OR "older adult*"[Title/Abstract] OR "senior*"[Title/Abstract] OR "retire*"[Title/Abstract] OR "geriatric*"[Title/Abstract]) AND ("Nursing Home"[Title/Abstract] OR "Nursing Homes"[MeSH Terms]) AND ("Frailty"[Title/Abstract] OR "Frailty"[MeSH Terms] OR “Frail Elderly”[MeSH Terms])

Frailty major mesh resulted in 750 articles

("Aged"[MeSH Terms] OR "aged, 80 and over"[MeSH Terms] OR "elder*"[Title/Abstract] OR "older people"[Title/Abstract] OR "older adult*"[Title/Abstract] OR "senior*"[Title/Abstract] OR "retire*"[Title/Abstract] OR "geriatric*"[Title/Abstract]) AND ("Nursing Home"[Title/Abstract] OR "Nursing Homes"[MeSH Terms]) AND ("Frailty"[Title/Abstract] OR "Frailty"[MaJR] OR "Frail Elderly"[MaJR])

Major mesh for nursing homes

("Aged"[MeSH Terms] OR "aged, 80 and over"[MeSH Terms] OR "elder*"[Title/Abstract] OR "older people"[Title/Abstract] OR "older adult*"[Title/Abstract] OR "senior*"[Title/Abstract] OR "retire*"[Title/Abstract] OR "geriatric*"[Title/Abstract]) AND ("Nursing Home"[Title/Abstract] OR "Nursing Homes"[MaJR] OR "Homes for the Aged"[ MaJR] OR "Long-Term Care"[ MaJR] OR "Residential Facilities"[MaJR])

majr Aged

(("Aged"[MaJR] OR "aged, 80 and over"[MaJR] OR "elder*"[Title/Abstract] OR "older people"[Title/Abstract] OR "older adult*"[Title/Abstract] OR "senior*"[Title/Abstract] OR "retire*"[Title/Abstract] OR "geriatric*"[Title/Abstract]) AND ("Nursing Home"[Title/Abstract] OR "Nursing Homes"[MeSH Major Topic] OR "Homes for the Aged"[MeSH Major Topic] OR "Long-Term Care"[MeSH Major Topic] OR "Residential Facilities"[MeSH Major Topic])) AND ((2010:2022[pdat]) AND (english[Filter]))

Residential Facilities

Long-term care facilities which provide supervision and assistance in activities of daily living with medical and nursing services when required.

Year introduced: 1968

# Homes for the Aged

Geriatric long-term care facilities which provide supervision and assistance in activities of daily living with medical and nursing services when required.

Year introduced: 1968

# Long-Term Care

Care over an extended period, usually for a chronic condition or disability, requiring periodic, intermittent, or continuous care.

Year introduced: 1992

# Nursing Homes

Facilities which provide nursing supervision and limited medical care to persons who do not require hospitalization.

PubMed search builder options

Final Search


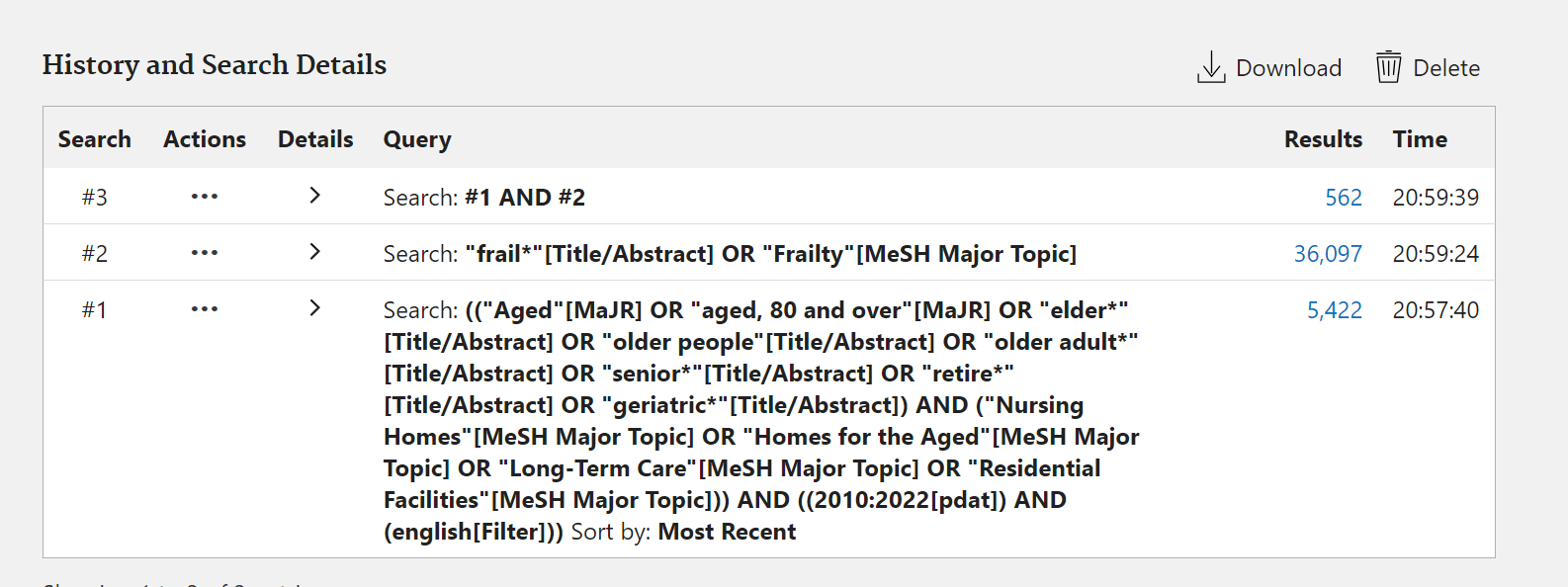


#1. Aged [MaJR]

#2. “aged 80 and over” [MaJR]

#3. ”elder*”[Title/Abstract]

#4. “older people*”[Title/Abstract]

#5. ”older adult*”[Title/Abstract]

#6. “senior*”[Title/Abstract]

#7. “retire*”[Title/Abstract]

#8. ”geriatric*”[Title/Abstract]

#9. #1 OR #2 OR #3 OR #4 OR #5 OR #6 OR #7 OR #8

#10. “frail*”[Title/Abstract]

#11. “Frailty”[MaJR]

#12. #10 OR #11

#13. “Nursing homes”[MaJR]

#14. “Homes for the Aged” [MaJR]

#15. “Long-Term Care” [MaJR]

#16. “Residential Facilities” [MaJR]

#17. #13 OR #14 OR #15 OR #16

#18. #9 AND #12 AND #17

#19. Limit #18 to English language

#20. Limit #19 to 2010-2022
